# Advances on inorganic scintillator-based optic fiber dosimeters

**DOI:** 10.1186/s40658-020-00327-6

**Published:** 2020-10-06

**Authors:** Liang Ding, Qiong Wu, Qun Wang, Yamei Li, Richard M. Perks, Liang Zhao

**Affiliations:** 1 School of Engineering, Cardiff University, Cardiff, UK; 2 Department of Pharmacy, General Hospital of Southern Theatre Command, Guangzhou, China; 3 Department of Pharmacy, Shanghai Baoshan Luodian Hospital, Shanghai, China; 4 Institute for Translational Medicine Research, Shanghai University, Shanghai, China

**Keywords:** Inorganic scintillators, Optical fiber dosimeters, In vivo, Real time dosimetry

## Abstract

This article presents a new perspective on the development of inorganic scintillator-based fiber dosimeters (IOSFDs) for medical radiotherapy dosimetry (RTD) focusing on real-time in vivo dosimetry. The scintillator-based optical fiber dosimeters (SFD) are compact, free of electromagnetic interference, radiation-resistant, and robust. They have shown great potential for real-time in vivo RTD. Compared with organic scintillators (OSs), inorganic scintillators (IOSs) have larger X-ray absorption and higher light output. Variable IOSs with maximum emission peaks in the red part of the spectrum offer convenient stem effect removal. This article outlines the main advantages and disadvantages of utilizing IOSs for SFD fabrication. IOSFDs with different configurations are presented, and their use for dosimetry in X-ray RT, brachytherapy (BT), proton therapy (PT), and boron neutron capture therapy (BNCT) is reviewed. Challenges including the percentage depth dose (PDD) deviation from the standard ion chamber (IC) measurement, the angular dependence, and the Cherenkov effect are discussed in detail; methods to overcome these problems are also presented.

## Introduction

As a fundamental process for RT treatment, RTD ensures that the radiation dose is safely and correctly delivered to the malignant abnormalities. However, current RTD practices have fallen behind the fast rapid scientific and clinical development of RT. Novel techniques applied to RT treatment include intensity-modulated RT (IMR), stereotactic ablative RT (SABR), and magnetic resonance imaging guided linear accelerator (MRI-linac). While these techniques provide better treatment opportunities for cancer patients, they also increase the complexity of the measurement of the dose rate and accumulated dose during treatment and are impacted by the limitations of existing dosimeters. Current safety and quality protocols are well established for traditional RT treatment techniques [1–4], but these protocols face challenges from aforementioned fast developing RT technologies. There are reports of patients receiving incorrect doses while undergoing radiation treatment utilizing these new RT techniques [5, 6]. This has aroused concerns over the lag of dosimetric equipment development behind the treatment techniques [7–9]. Therefore, developing radiation dosimeters capable of real-time in vivo dose monitoring has received significant research attention.

MOSFETs and diode-based devices have been utilized for clinical real-time in vivo dosimetry [10–12] owning to their good sensitivity, small size, and real-time readout capabilities. However, MOSFETs are expensive, incident angle-dependent, and of limited durability. Diode dosimeters are sensitive to accumulated dose, temperature, and incident angle. SFD provides an alternative solution for real-time in vivo dosimetry. It is a relatively new type of detector that involves coupling of a scintillator, an optical fiber, and a light-detector. The combination of the scintillator and the optical fiber endows SFD attractive merits such as passive detection, small size, linear response to dose rate, energy independence, immunity to the electromagnetic interference, good mechanical robustness, and capacity for multiplexing. Though the clinical applications of SFD are limited, they have shown great potential for real-time in vivo dosimetry.

To the best of our knowledge, the ideal of a “SFD” first emerged in 1969 when Byfield et al. [13] reported a plastic scintillator-based optical fiber dosimeter for intracavitary dosimetry. The probe comprising a piece of plastic phosphor encapsulated in a stainless-steel tube. The optical signal was collected and transmitted via a Lucite “light pipe”. The detector achieved good accuracy measuring the radiation dose from cobalt^60^ and megavoltage X-rays in a phantom. Although this probe dosimeter might appear primitive and too large for actual real-time in vivo dosimetry, SFDs later reported/shared the similar design which combines a small piece of scintillator, an optical fiber, and a photodetector.

The fast development of SFD emerged in the 1990s due to seminal and systematic research on plastic scintillator optical fiber dosimeters (PSFDs) [14, 15]. Since then, SFDs have attracted more and more research attention, and numerous articles investigated aspects of SFDs such as scintillator materials [16, 17], configuration optimization [18, 19], and signal processing techniques (e.g., Cherenkov effect removal) [20, 21]. Several featured review papers on SFD development exist in the scientific literature as well [8, 9, 22–26].

The scintillation materials applied in SFDs can be classified into two broad groups. One group is the plastic OS comprising aromatic hydrocarbon molecules. They have similar mass and electron densities as those of water and human tissue. Thus, they have photon/electron interaction properties similar to water and human tissue (i.e., “water-equivalent” property). Another group includes the IOSs, most of which are alkali halide (e.g., NaI:Tl, CsI:Tl, and CsI:Na) or oxide (e.g., Y_2_O_3_:Eu and Lu_2_O_3_:Eu) crystals doped with lanthanide ions. Though IOSs have been widely utilized for X-ray detection and imaging, their application in SFD is still quite limited partially due to their non-linear response to X-ray energies under 100 keV and non-water equivalency.

However, research on the IOS-based SFDs (IOSFDs) shows that this particular type of SFD has promise in RT dosimetry applications due to their high sensitivity to low radiation dose rate and overwhelming signal intensity compared with the Cherenkov effect. This paper reviews the state-of-the-art development of IOSFDs for radiation dosimetry in oncology, focusing on X-ray RT. We first evaluate the fundamentals of IOSFD in the “Fundamentals” section. The scintillation mechanism of scintillators under ionizing photon radiation will be briefly introduced—this section evaluates the properties of the scintillators that we considered important for RT dosimetry (RTD). Second, we look at the development of IOSFDs using different scintillators and designs in the “Development of IOSFDs” section including their applications for dosimetry in RT such as BT, PT, and BNCT. Third, we discuss some challenges facing IOSFD for RT dosimetry application in the “Challenges of IOSFD for RTD application” section including the measured PDD discrepancy in comparison with the standard IC measurement and the angular dependence. The Cherenkov removal techniques are summarized in the “Cherenkov effect removal techniques” section. Finally, we conclude the article and provide a future outlook of ISFD for RTD.

## Fundamentals

**Fig. 1:**
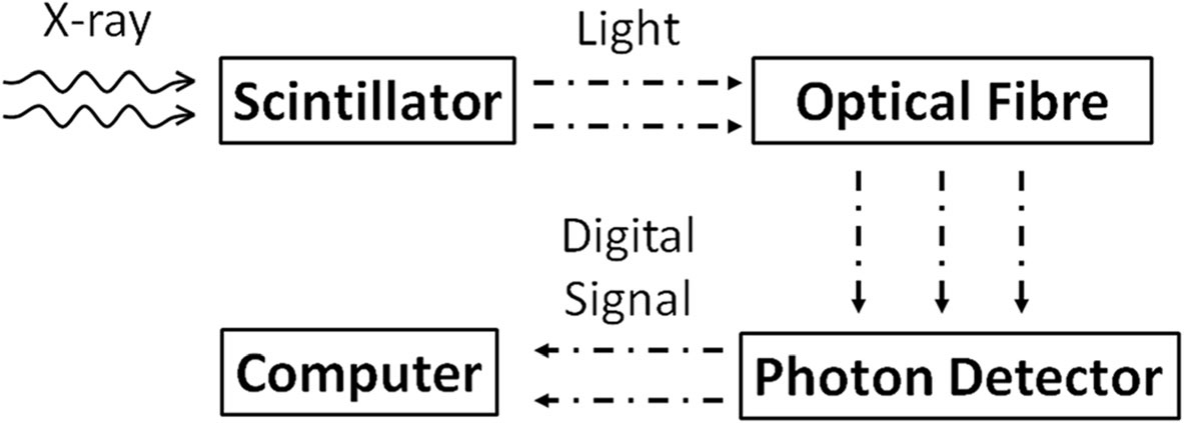
A simple schematic of the radiation detection process using SFD

The regular SFD configuration involves the coupling of a piece of scintillation material to an optical fiber. Figure 1 gives a simple schematic of how SFD works under the ionizing radiation. The scintillator undergoes a radio-luminescence process when irradiated by a high-energy ionizing beam (Fig. 1). The light photons generated in the scintillator are collected by an optical fiber and remotely transmitted to the photon detector (e.g., photomultiplier, CCD camera, and spectrometer). The output can be analyzed via a remote computer terminal. Theoretically, the scintillation light emission is proportional to the dose rate.

Overall, the performance of the SFD for oncology radiation measurement is determined by the scintillation light collection efficiency and the response of the scintillator to the incident radiation particles. The scintillation light collection efficiency resembles the light extraction efficiency for LED. It describes how efficiently the scintillation light is extracted and transported by the optical fiber coupled to the scintillator. The scintillation light photons are emitted towards arbitrary directions. To collect these photons as many as possible, it is key to achieve an efficient coupling between the scintillation domain and the optical fiber with a limited numerical aperture. Such coupling required a delicate design of the configuration of the IOSFD, and this issue will be reviewed and discussed later in the “Development of IOSFDs” section.

The response of the scintillator to the radiation is determined by several factors. Taking RTD with an X-/γ-ray radiation source for examples, these factors include the size or the concentration of the scintillation material, the X-ray linear attenuation coefficient, the scintillation rise-decay time, and the overall radiation-to-light conversion efficiency. Focusing on dosimetry for ionizing photon radiation, we next briefly introduce the scintillation mechanism and then look at several physical properties of scintillators that are considered important for IOSFD.

### Scintillation mechanism

IOSs normally have broad band gaps where electrons can jump up to higher energy levels via excitation once absorbing ionizing radiation energy. The excited electrons can also drop down to lower energy levels via de-excitation through the emission of (visible) photons. The scintillation process under ionizing X-ray radiation can be divided into three stages: (a) primary photon interaction, ionization of atoms, relaxation, and thermalization of secondary particles; (b) transport of electrons and holes and further relaxation; and (c) luminescence [27, 28]. The attenuation of X-ray photons takes place in stage (a) in the form of four possible primary photon interactions, i.e., photoelectric absorption, Compton scattering, electron-positron pair production, and coherent scattering. Some of the energy absorbed by the scintillator will generate free electron holes; the other is transferred to a lattice vibration (i.e., heat) or secondary X-rays or electrons radiation. In stage (c), the electrons and holes are trapped by the luminescence centers (defects or dopant activators, e.g., Mn^2+^, Sn^2+^, Eu^3+^, and Ti^4+^), which contributes to radiative recombination in the form of ultraviolet/visible (UV/vis) light emission. For in vivo or under-surface phantom dosimetry, the scintillation material is excited not only by direct X-ray radiation, but also by secondary electrons generated via Compton scattering or photoelectric interaction in the tissue-equivalent material.

### Basic physical properties of scintillators

Generally, the IOS for photon radiation detection should have a high light yield, high X-ray linear attenuation coefficient, short scintillation-decay time, low afterglow, good radiation resistance, and linear-response with incident X-ray or electron beams. Due to the long history and consistent research efforts concerning developments in this area, there are numerous types of scintillators available. Many articles and books have systematically reviewed and illustrated the work conducted on scintillator development and the application of scintillators for radiation detection [26, 28–34]. The synthesis and characterization of scintillators are beyond the scope of this review, and this work briefly introduces the physical parameters describing the scintillator with respect to inorganic scintillation materials used in existing reported IOSFDs [35–37].

The parameters describing the scintillation efficiency include the light yield (*N*_ph_/*E*_*γ*_) and the overall scintillation efficiency *η*. (*N*_ph_/*E*_*γ*_) for photons describes the number of UV/vis photons (*N*_ph_) produced in the scintillation conversion per energy *E*_*γ*_ (usually in MeV) of incoming X-/γ-ray [27]. The overall scintillation efficiency *η* is usually used to evaluate the scintillation ability of X-ray phosphors, and it is the ratio of the energy emitted as scintillation light and the energy absorbed by the scintillation material [38]

1 $$ \eta ={N}_{\mathrm{ph}}\bullet {E}_{\mathrm{vis}}/{E}_{\gamma } $$

where *E*_vis_ is the energy of generated UV/vis photons.

The X-ray linear attenuation coefficient (*μ*, cm^−1^) describes how fast the X-ray photon is attenuated in a material. It is determined by the mass-density *ρ*, the effective atomic number (*Z*_eff_), and the ionizing photon energy *E*. Normally, dense IOSs comprising heavy elements (e.g., Gd, Lu, and I) have relatively higher value of *μ* than those of OSs, as is shown in Table 1. Term *μ* is the sum of the linear attenuation coefficients of the primary photon interactions *μ*_i_ (“i” represents “photoelectric,” “Compton,” or “pair”), given by:

2 $$ {\displaystyle \begin{array}{c}\mu ={\mu}_{\mathrm{photoelectric}}+{\mu}_{\mathrm{Compton}}+{\mu}_{\mathrm{pair}}\\ {}=\left[{\left(\frac{\mu }{\rho}\right)}_{\mathrm{photoelectric}}+{\left(\frac{\mu }{\rho}\right)}_{\mathrm{Compton}}+{\left(\frac{\mu }{\rho}\right)}_{\mathrm{pair}}\right]\bullet \rho =\frac{\mu }{\rho}\bullet \rho \end{array}}\kern0.5em $$

**Table 1:** The physical properties of typical IOSs compared with OSs BCF-10 and BCF-12 [28, 30, 34, 39–42]

Scintillator	Density (g∙cm^−3^)	Efficiency *η* (%)	Light yield photons/MeV	Emission maximum (nm)	Decay time (ns)
Gd_2_O_2_S: Tb	7.3	16	60,000	540	6 × 10^5^
Gd_2_O_2_S: Pr	7.3	15	56,000	513	~ 7 × 10^3^
Gd_2_O_2_S: Eu	7.3	12	45,000	626	~ 10^6^
NaI:Tl	3.67	11.3	41,000	410	230
CsI:Tl	4.51	13.7	66,000	550	800
ZnS:Ag	3.9	17–20	NA	450	~ 1000
Y_2_O_3_:Eu	5.0	10	NA	611	~10^6^
Y_2_O_2_S:Eu	4.89	12	NA	670	6 × 10^5^
YVO_4_:Eu	4.22	7	NA	625	8 × 10^5^
Y_3_Al_5_O_12_:Ce	4.56	3–5	24,000	550	90–120
ZnWO_4_	7.62	NA	9500	490	2 × 10^4^
BCF-10	1.05	NA	~ 8000	432	2.7
BCF-12	1.05	NA	~ 8000	435	3.2

where ${\left(\frac{\mu }{\rho}\right)}_{\mathrm{photoelectric}}$, ${\left(\frac{\mu }{\rho}\right)}_{\mathrm{Compton}}$, and ${\left(\frac{\mu }{\rho}\right)}_{\mathrm{pair}}$ are the mass partial interaction coefficients of the three primary photon interactions; *μ*/*ρ* is the total mass interaction coefficient. The behavior of photon beam (flux intensity is *I*) attenuation when transporting a distance *l* in a uniform material is usually described by the exponential law [43]

3 $$ I(l)={I}_0\bullet \exp \left(-\mu \bullet l\right) $$

where *I*_0_ is the initial flux intensity.

**Fig. 2:**
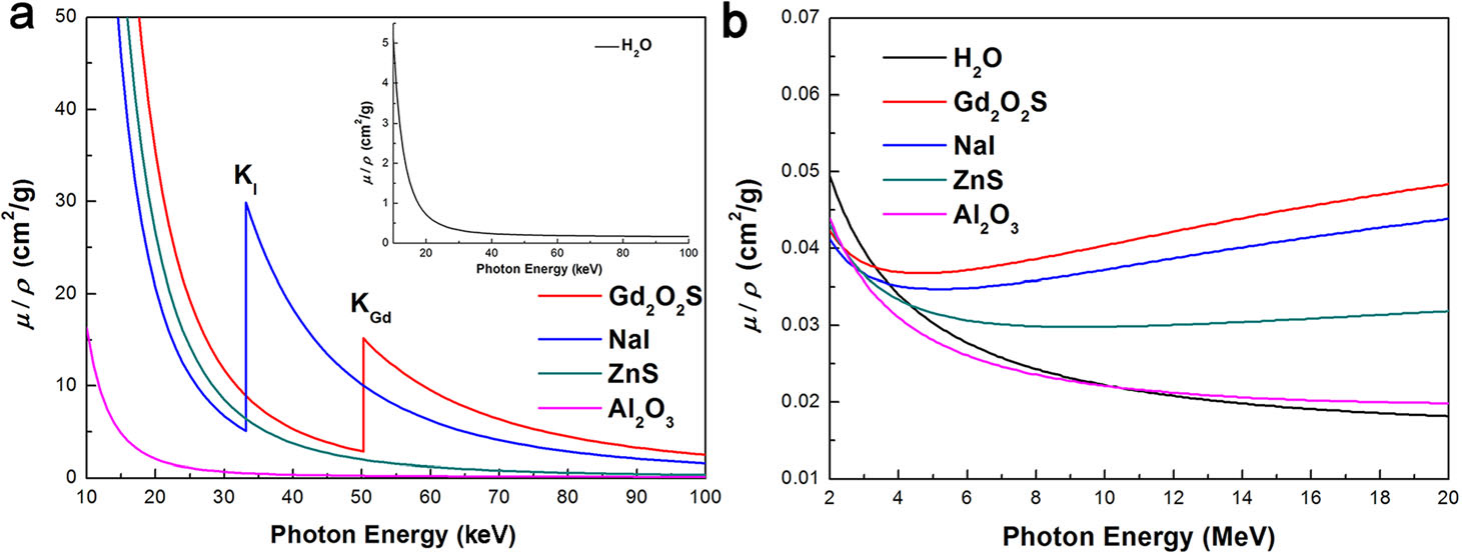
The photon mass attenuation coefficients *μ*/*ρ* (*g* ∙ *cm*^−3^) of IOSs Gd_2_O_2_S, NaI, ZnS, and Al_2_O_3_ for photons in the energy ranges 10–100 keV (**a**), and 2—22 MeV respectively. The insert in (**a**) is the mass attenuation coefficient of H_2_O

The total mass attenuation coefficients (*μ*/*ρ*) of some IOSs used for reported IOSFD construction or applications are shown in Fig. 2, which are sourced from the XCOM database [44]. For ionizing photons with relatively low energies under 100 keV, photoelectric absorption is the dominant type of photon interaction for IOSs. The energy of the incident photon is totally absorbed during this process. For the water-equivalent material, Compton scattering is the main type of photon interaction when the photon energy is larger than 30 keV [45], and only part of the photon energy is absorbed for light signal generation. As for γ-rays of high energy (more than a few MeVs, as shown in Fig. 2b) used for RT treatment, Compton scattering and electron-positron pair production are predominant, and (*μ*/*ρ*) for γ-rays decreases distinctively compared with X-rays in low energy ranges (Fig. 2). More information regarding this topic is published [43, 46, 47]. Due to different photon interaction mechanisms, higher values of (*μ*/*ρ*), and *ρ*, most IOSs exhibit distinctively higher X-ray absorption coefficients than those of OSs in both low (< 100 keV) and high (> 2 MeV) photon energy ranges. We note here that the energy mainly refers to the energy level of incident X-rays, which is determined by the accelerating voltage of the X-ray tube or linac.

The mass attenuation coefficients (*μ*/*ρ*) of most materials decreases with respect to photon energy in 10–100 keV energy range (Fig. 2a). However, for scintillators comprised of heavy elements such as Gd or I, there is an abrupt rise in the (*μ*/*ρ*) − *E* curve, owning to the characteristic photoelectric absorption at K-edge (e.g., I: 33.17 keV; Gd:50.02 keV). The relationship between (*μ*/*ρ*) and *E* may explain the energy-dependent sensitivity of IOSFD in low-energy X-ray radiation.

**Fig. 3:**
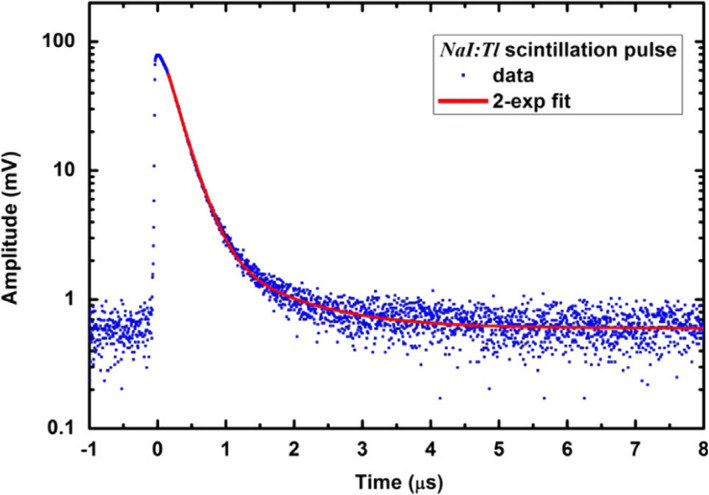
Averaged scintillation pulse recorded for NaI:Tl irradiated with a ^137^Cs source [48]. Two exponential terms fit to the data are presented with solid line

The scintillation decay time relates to the scintillation kinetics. The “Scintillation mechanism” section has introduced the scintillation mechanism. The photon absorption and generation of e^−^-h^+^ pairs are instant; thus, the emission time is determined by the relaxation, thermalization, and transport of e^−^-h^+^ pairs and the luminescence process. Taking NaI:Tl for example, the time profile of the scintillation is shown in Fig. 3 [48]. Once irradiated, the scintillation pulse rises quickly to the top with a typical rise time within 1 ns. The decay time, however, can be as slow as a few nanoseconds and as high as several milliseconds. The decay can be approximated using an exponential [28]

4 $$ I(t)\sim \exp \left(-t/\tau \right) $$

where *τ* is the decay time. The decay time of NaI:Tl is shown in Fig. 3 as about 230 ns. Rare-earth elements (e.g., Tb, Pr, and Eu) are often introduced as dopants into IOSs and normally act as luminescence centers. The decay time will also change accordingly with respect to the dopants. Table 1 shows that with the same matrix of Gd_2_O_2_S, the scintillator doped with europium (Eu) exhibits the longest decay time (*τ* ~ 10^6^ ns). Gd_2_O_2_S with the dopant praseodymium (Pr) has the shortest decay time (*τ* ~ 7 μs). This is because the allowed 5*d* → 4*f* transitions of Pr^3+^ ions (the luminescence centers) are fast (tens of nanoseconds), while the lifetime of forbidden and faint *f* → *f* transitions (^5^*D*_0_ → ^7^*F*_j_) of Eu^3+^ is much slower. To achieve real-time dosimetry, the IOSs chosen for IOSFD should have decay time that is short enough so as to secure accuracy, due to its effect on the sampling frequency.

Normally, it is required that scintillator material used for real-time monitoring has short decay time. However, the long decay time of some scintillators relative to the Cherenkov effect has also inspired solutions for eliminating the Cherenkov interference. For example, the BC-444 scintillator (Saint-Gobain Crystals) was chosen by Archer et al. [49] to fabricate a PSFD because of its slow rise and decay time compared to the Cherenkov effect. They applied an algorithm-based temporal Cherenkov removal technique to eliminate Cherenkov interference. The decay time of NaI:Tl is similar to that of BC-444 (285 ns). Other inorganic scintillators such as Gd_2_O_2_S:Tb and YVO_4_:Eu have much longer decay time; thus, the same temporal Cherenkov removal technique can also be applied when using IOSFD.

IOSs with high light yields, e.g., Gd_2_O_2_S:Tb and CsI:Tl, have already shown good performance in X-ray computed tomography. The high X-ray linear attenuation coefficient and overall scintillation efficiency endow IOSs with great potential in low dose detection application. Moreover, numerous scintillators or phosphors with maximum emission peaks in red to infrared wavelength range provide convenience for the stem effect removal. However, there is still significant concern over IOS applied for real-time dosimetry. In the following sub-sections, the factors that limit the IOSFD application in medical radiation dosimetry are presented along with the advantages of utilizing IOSs for SFD dosimetry.

## Development of IOSFDs

To the best of our knowledge, Swinth et al. [50] first applied IOSFD for low-background, low-energy biomedical photon counting. The biomedical radiation-sensitive probe comprised a large piece of NaI : Tl crystal coupled to a bundle of glass optical fibers. The probe exhibited a higher sensitivity to low-energy ^239^Pu photons (17 keV, 60 keV) than the diode dosimeter developed for the same purpose.

**Fig. 4:**
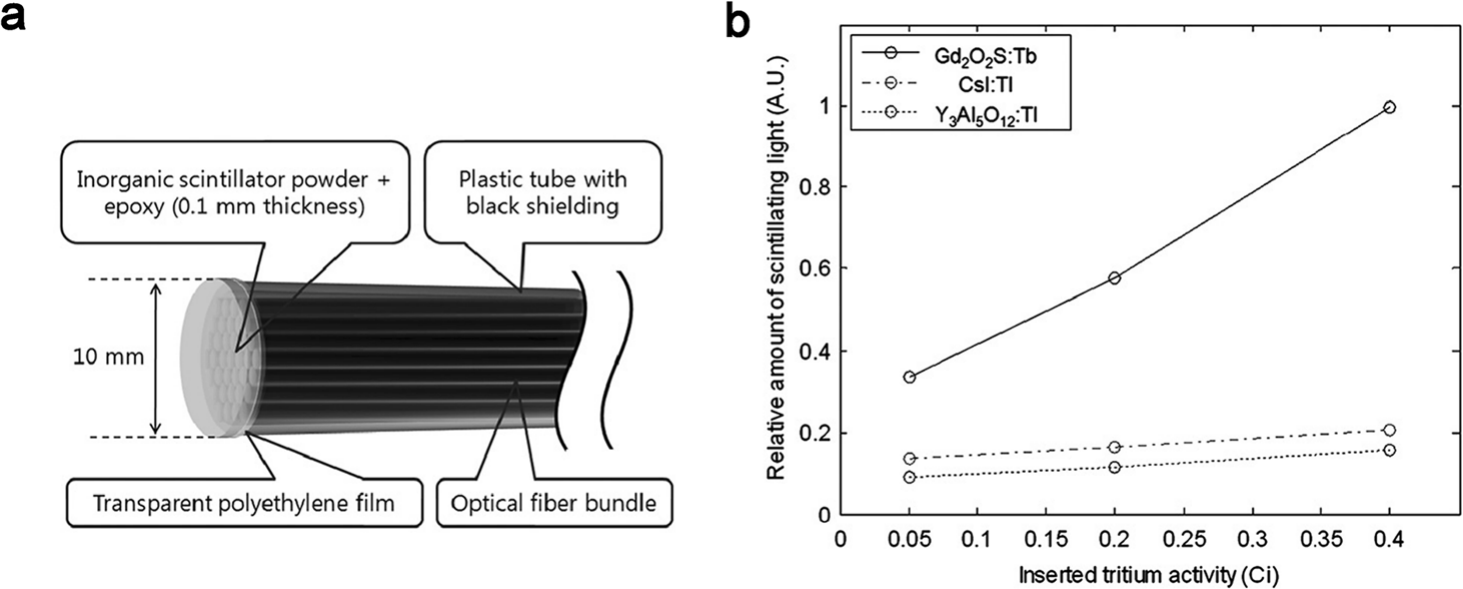
IOSFD for detection of tritium [51]. **a** A schematic diagram of sensor tip. **b** Measured amounts of scintillation photon with three kinds of IOS

Besides ionizing X-/γ- ray detection, research efforts have also been paid to explore the potential of IOSFDs for the dosimetry of biomedical particle radiations. For example, Jang et al. [51] compared the *β*-ray (electrons) detection ability of different scintillator-coupled optical fiber dosimeters. As shown in Fig. 4a, the sensor tip is fabricated using IOSs and plastic optical fiber bundles. With a metal hydride type of tritium source, three kinds of scintillators (Gd_2_O_2_S:Tb, Y_3_Al_5_O_12_:Ce, and CsI:Tl) were used for sensor fabrication to select the best scintillator. Among these three scintillators, the IOSFD with Gd_2_O_2_S:Tb exhibited the best scintillation response in terms of generated photons (Fig. 4b).

In the recent decades, IOSFDs using various kinds of scintillators and configurations were developed, and their potential application for RTD such as external RTD, small-field in-vivo dosimetry, and BT has been investigated. In the following sections, the research on IOSFDs for X-ray RTD will be reviewed; efforts of utilizing IOSFDs for BT dosimetry, PT, and BNCT would also be concluded.

### IOSFD for X-ray RTD

**Fig. 5:**
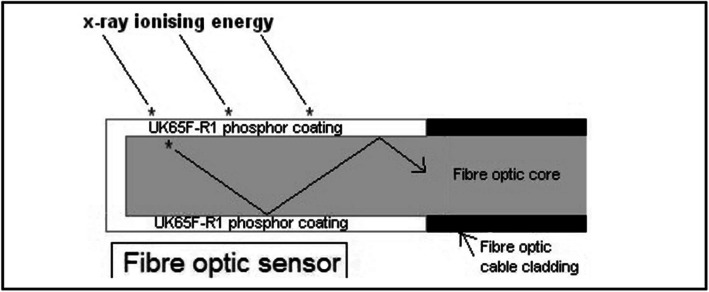
The fiber optic radiation dosimeter sensor design reported by McCarthy et al. [52]

A IOSFD based on Gd_2_O_2_S:Tb was presented by McCarthy et al. [37, 52]. The schematic illustration of the probe structure is shown in Fig. 5. The cladding layer of the PMMA fiber tip was removed, and the scintillator powders were coated surrounding the fiber core using an epoxy resin-based molding method. Though this dosimeter was less sensitive to X-ray energy below 50 kVp, it exhibited good sensitivity, stability, and repeatability of measurement for various levels of low-energy ionizing X-ray (50–140 kVp). The dosimeter also demonstrated excellent spectral response and repeatability for γ-ray radiation as high as 6 MV and 15 MV. This research carried out an initial characterization measurement of IOSFD in both air and water phantom and is a useful study of IOSFD concerning the linearity, repeatability, stability, and effective coupling between scintillator powders and optical fiber. However, it would still need more research effort into the angular dependence, radiation field-size dependence, and the PDD measurement, for a more comprehensive assessment of this dosimeter for real-time in vivo dosimetry.

**Fig. 6:**
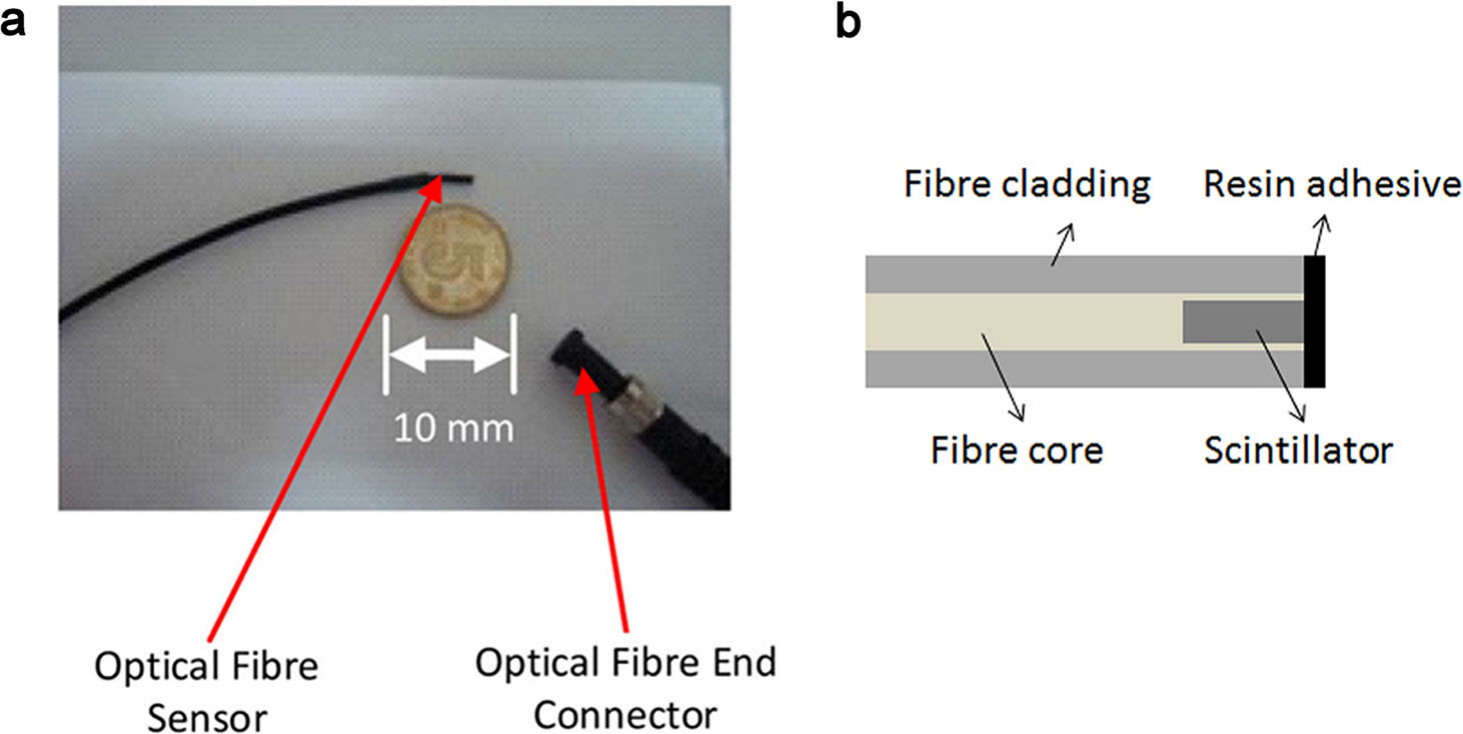
Embedded structure of an IOSFD. **a** The IOSFD (photograph) presented by O’Keeffe et al. [53]. **b** The IOSFD (schematic representation)

The coupling efficiency between the scintillator and the optical fiber is important for the light collection. To improve the coupling efficiency, O’Keeffe et al. [53] proposed a new design by embedding a piece of Gd_2_O_2_S:Tb scintillator into the core of an optical fiber (Fig. 6). This design was further confirmed and investigated by Qin et al. [19]. The configuration not only reduced the transmission loss, but also isolated the scintillator from the surrounding environment. Except from the excellent signal-to-noise ratio, linearity, and repeatability, the detector showed a great isotropic response to both radical and axial changes of the incident radiation as well.

**Fig. 7:**
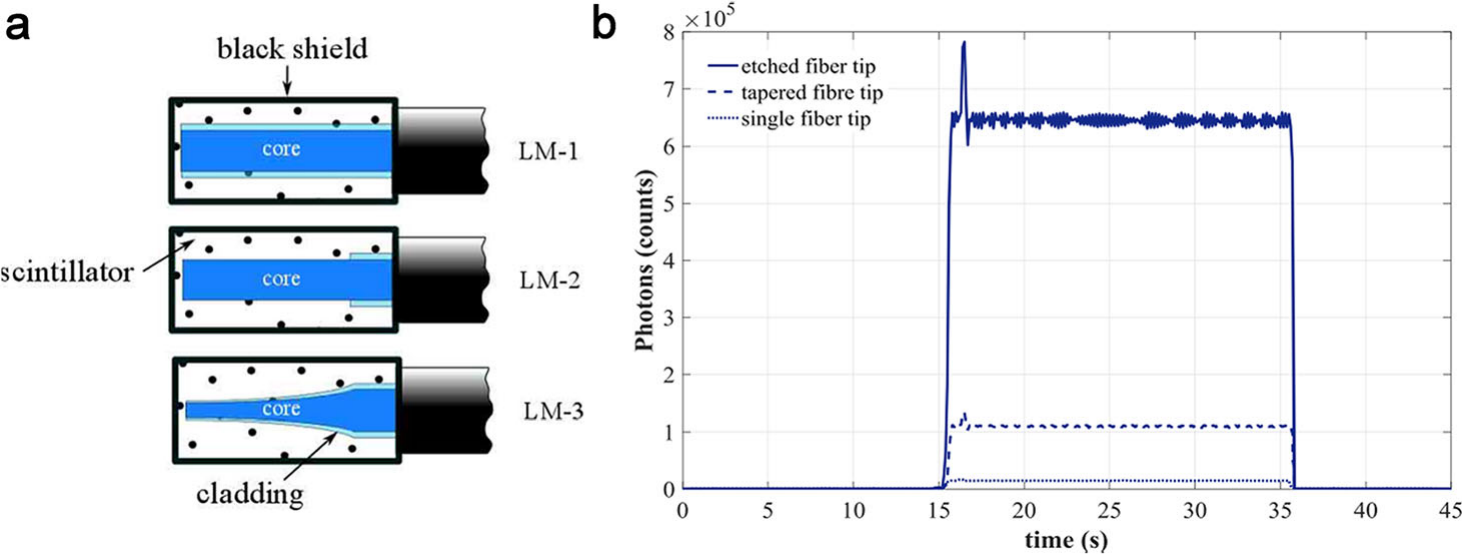
**a** Schematic draw of the fabricated transducers LM-1 (plain fiber), LM-2 (etched fiber), and LM-3 (tapered fiber). **b** Sensor signal recorded for single fiber tip (dotted line), tapered fiber tip (dashed line), and etched fiber tip (solid line) [54]

Another effort to increase the light coupling efficiency between the scintillation domain and optical fiber of Gd_2_O_2_S:Tb IOSFD was presented by Andres et al. [54]. The basic idea of the sensor configuration optimization is accelerating the light extraction efficiency from scintillation domain via expanding the useful surface in contact with the scintillator. The sensor tip (Fig. 7) is modified by either chemical etching (LM2 in Fig. 7a) or thermomechanical tapering (LM3 in Fig. 7a). Precautions to minimize the air-bubble density were also taken, aiming to increase the optical signal intensity. All fabricated devices have been tested under X-ray irradiation (6 MV) from a linac at a dose rate of 300 monitor units/min with a readout every 100 ms. The results of LM2 (chemical etching) shown in Fig. 7b demonstrated a signal improvement of up to 43 times compared with the previously reported proposals using IOSs and polymer fibers (LM1). Moreover, a scintillator format comparison between scintillator powders and scintillator-epoxy mixture demonstrated that the IOS used in powder format could achieve higher sensitivity. This is due to the higher packing density of the scintillator in the scintillation domain. This is another successful illustration of improving the light coupling efficiency between the scintillator and optical fiber through sensor tip modification.

Research on ruby-based IOSFD was reported by Jordan et al. in 1996 [35]. Here, a ruby (Al_2_O_3_:Cr, 1 mm in diameter)-coupled PMMA optical fiber was used to evaluate the integrated dose of both X-ray and electron beams generated with Varian CL600C and 2100A medical linacs. The time-delayed method combined with optical filtering method was used to remove the stem effect (Cherenkov effect and fluorescence) signal from the optical fiber. The results demonstrated that this time-delayed gated signal integration method is effective for eliminating the fluorescent and Cerenkov light origination in the irradiated optical fiber. Irradiated by 4 MV photon beams and 9–12 MeV electron beams, the PDD measurements with this probe agree well with values using an ionization chamber. Therefore, it is possible to apply ruby-based IOSFD for radiation dosimetry. This temperature dependence of Al_2_O_3_:Cr was also investigated in this study, and a temperature correction on the order of 0.6% per degree was suggested for clinical application.

The application of Al_2_O_3_:Cr ruby-based IOSFD in radiotherapeutic field was further investigated by Teichmann et al. [55]. A ruby (Al_2_O_3_:Cr) crystal (1-mm diameter) was adhered to a fused silica light guide (BFH-400, Thorlabs) with optically transparent epoxy (EPO-TEK® 301-2, Epoxy Technology). An optical single-band pass filter (Semrock 689/23 nm BrightLine®) was adopted to remove the unwanted stem signals from the fiber guide. The probe was tested under irradiation of both electron and photon radiation with a radiation dose up to 0.5 kGy. The radioluminescence signal shows a slight linear rise with an accumulated dose up to 2 Gy, which is stable for large doses up to 0.5 kGy. The sensor also demonstrated good sensitivity to dose as low as 2.6 ± 0.2 mGy/h. The stem effect produced in optical fibers covers a wide range of wavelengths [20, 56], and it cannot be completely eliminated. Thus, the stem signal below and above the energy threshold for the generation of Cherenkov radiation could be modeled by a linear function according to their research.

Yttrium phosphors doped with Eu such as Y_2_O_3_:Eu, Y_2_O_2_S:Eu, and YVO_4_:Eu emit bright red-light under UV or X-ray excitation; these have been used widely in the industry (e.g., white LEDs). Attempts have also been made to utilize them for dosimetry [57–60]. One merit of these Eu-doped yttrium phosphors is that they provide the convenience of the stem effect removal with the simplest optical-filtering method. The stem effect (including Cherenkov effect and fluorescence of the plastic optical fiber) dominates in the blue/green spectral region; therefore, combining a red-emission phosphor with a long-pass optical filter can effectively suppress or remove these spurious signals [61, 62].

**Fig. 8:**
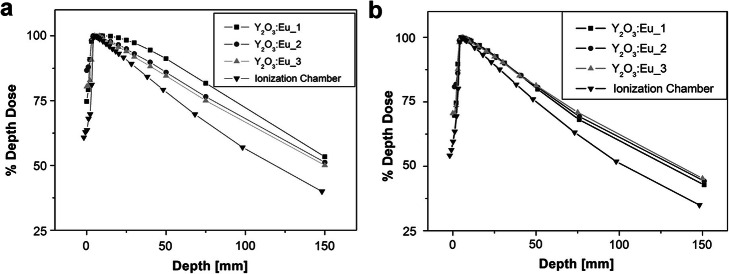
The PDD curves from Y_2_O_3_:Eu-based FOD compare to that of a standard IC under ^*60*^Co irradiation. Surface to source distance is 80 cm and the irradiation field is set to **a** 10 × 10 *cm*^2^ and **b** 5 × 5 *cm*^2^ respectively [58]. Different phosphor coating thickness is applied

Molina et al. investigated the feasibility of utilizing Eu-doped yttrium phosphors for fiber optic dosimetry [57]. The radio-luminescence response of three commercial phosphors (Y_2_O_3_:Eu, Y_2_O_2_S:Eu, and YVO_4_:Eu) was characterized. All three materials showed no changes in RL response as dose accumulates with no afterglow decay. The same research group further studied the feasibility of using Y_2_O_3_:Eu-based IOSFD for real-time dosimetry under ^60^Co irradiation [58]. The stem effect signal was successfully suppressed via a long-pass optical filter (610 nm). Figure 8 shows that the PDD profile of the IOSFD was affected not only by the irradiation field size but also by the coating thickness of the Y_2_O_3_:Eu phosphor. The cause of this PDD deviation was attributed to the higher atomic number of Y_2_O_3_:Eu in this research. The larger proportion of phosphor acts as a scintillator with respect to the larger irradiation field size.

A more reasonable explanation, according to the research of Qin et al. [63], is that the inorganic scintillators are more sensitive to photons with energies under 1 MeV. Due to multiple Compton scattering events during the photon transporting in water, the photon energy keeps decreasing with respect to the increasing water depth (i.e., softening of the photon spectrum). The energy eventually reaches below 1 MeV where the photon electronic interaction occurs. As a result, the inorganic scintillator exhibits an over-response compared to IC and PSFD. Nevertheless, this result demonstrated the potential of Y_2_O_3_:Eu-based IOSFD for in vivo and real-time dosimetry; however, the coating thickness of the phosphor attached to the fiber is not given, and the angular dependence requires further illustration regarding this kind of dosimeter.

The consistent work on YVO_4_:Eu-based IOSFD was reported by Martínez et al. [59, 64]. A temporal discrimination method was applied to eliminate the stem effect. The Cherenkov effect is a fast process and decays rapidly without radiation; scintillation is a delayed process. Therefore, the temporal discrimination method can effectively remove the Cherenkov signal by using a scintillation material with relatively longer radio-luminescence lifetime [20]. In this case, a pulse radiation mode was set with the linac, and only the optical signal starting 20 μs after each linac pulse reaching the detector was recorded. The YVO_4_:Eu-based IOSFD demonstrated temperature-independence, good spatial resolution, and capability for real-time dosimetry. The azimuthal angle experiment test showed that the geometry of the detector determines angular-dependent response to the radiation [59]. The Monte-Carlo simulation method was successfully used to verify the PDD profiles measured with the IOSFDs and the standard IC in the fields with different sizes.

### IOSFD for BT dosimetry

To date, commercially available in-vivo dosimeters for BT can only measure the total dose post-treatment, which might introduce errors and uncertainties during the process. Given the small size, durability, and fast and linear response of PSFDs, there have been reports of using PSFD or PS-based radiation detector for in vivo BT dosimetry and source tracking to improve the dose delivery accuracy [65–68]. Compared to PS, IOSs are more sensitive to low dose radiation and have much higher scintillation efficiency; thus, IOS-based radiation detectors have great potential for in vivo BT dosimetry.

**Fig. 9:**
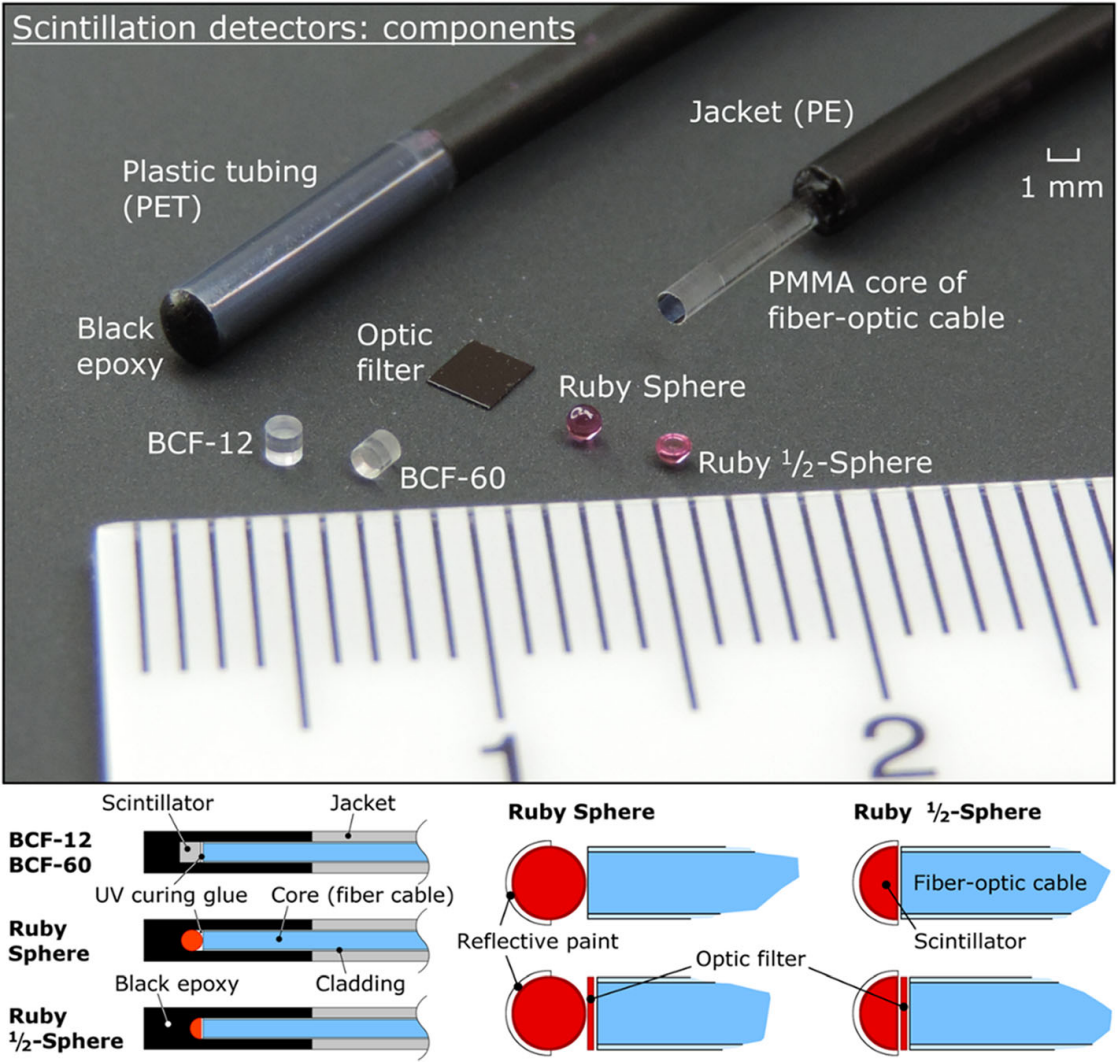
The 3 types of detector probe tips that were used in the experiments in ref [69]

Kertzscher and Beddar tested the potential of the Al_2_O_3_:Cr-based IOSFD for dosimetry in ^192^Ir BT [69]. This ruby-based IOSFD was compared with common PSFDs. The doping concentration of Cr^3+^ is 0.5%, and the OSs chosen are BCF-12 and BCF-60. Also, in this experiment, three types of IOSFD configurations were tested (Fig. 9): one without reflective paint and optical filter, one with reflective paint but no filter, and one with both a filter and reflective paint. The optical coupling effect of different ruby morphologies was investigated, namely, sphere and half-sphere. The air-kerma strength of the ^192^Ir source is between 17.6 and 40.0 mGy m^2^·h^−1^ throughout the experiments. The contribution of the stem effect and photoluminescence signal from the fiber was quantified. By incorporating a long pass filter between the scintillator and optical fiber, the unwanted photoluminescence from fiber was suppressed from 1 to 5% as the radiation source dwelled 0.5 cm away from the fiber-optic cable. The stem effect was suppressed as low as < 3% using a band-pass filter because the distance from the scintillator to the source is smaller than 7 cm. The IOS exhibited a much stronger scintillation signal than that of OSs—up to 20-fold that of BCF-12-based PSFDs. The result shows that by choosing IOSs with insignificant time-dependent luminescence and afterglow, the inorganic IOSFDs are promising candidates for in vivo high-dose-rate BT dosimetry.

In addition to the Al_2_O_3_:Cr ruby, Kertzscher and Beddar also studied four types of IOSs-based fiber dosimeters for real-time BT dosimetry [60, 70]. The scintillation property of commercial Eu-doped phosphors Y_2_O_3_:Eu, YVO_4_:Eu, Y_2_O_2_S:Eu, and Gd_2_O_2_S:Eu was assessed and compared to plastic scintillators BCF-12 and BCF-60. The IOSFD exhibited scintillation intensities about 16–134 times of that of BCF-12-based PSFD. More importantly, this article showed that the time-dependent response caused by the afterglow of Eu-doped scintillators can be suppressed by the mixture of Y_2_O_3_:Eu and YVO_4_:Eu with an appropriate weight ratio. The same research group later compared ruby (Al_2_O_3_:Cr), Y_2_O_3_:Eu, YVO_4_:Eu, ZnSe:O, and CsI:Tl-based IOSFDs for ^192^Ir BT and also used BCF-12 and BCF-60 as a reference [70]. According to their test, ZnSe:O-based IOSFD exhibited the most favorable characteristics considering its high and stable scintillation intensity, dose linearity, insignificant memory effects, and negligible stem interference. However, all IOSFDs tested are heavily energy dependent because of the non-water equivalent characteristic of IOSs and the distant-dependent energy spectra of ^192^Ir BT source in water. As a result, further attempts to acquire signal intensity-dependent correction factors are needed.

The possibility of doing time-resolved in vivo dosimetry with the IOSFD for source tracking in BT was also explored [67, 71, 72]. Belley et al. [71] adopted an inorganic nanocrystalline composition (Y_2_O_3_:Eu,-Li) as the scintillation part and connected it to a silica-based optical fiber. The clinical performance of real-time dose rate monitoring during high-dose-rate (HDR) BT was evaluated and compared to thermoluminescent dosimeters and the treatment planning system. The result conferred the feasibility of using Y_2_O_3_:Eu,-Li-based IOSFD for real-time measurements during HDR BT. Johansen et al. [67] used Al_2_O_3_:C-based IOSFD as an in vivo dosimeter for source tracking BT. The IOSFD was placed in a delicate BT needle in the prostate near a ^192^Ir radiation source. The delivered dose rate and total dose were recorded with a single IOSFD, and the distance between the source and dosimeter was derived from the measured dose rate. The shift of the treatment needle was successfully detected with the IOSFD. This work demonstrated the great application potential of IOSFD to source tracking in BT. The same research group later used the Al_2_O_3_:C-based IOSFD to verify the dwell time in HDR BT [72]. By analyzing the dose rate at different sampling rates, dwell times in the identified dwell positions were calculated with high accuracy. Though the detection efficiency needs to be improved, the research proposed an effective method to detect dwell time offsets in BT. Overall, IOSFD-based in vivo dosimetry has promising applications in BT dosimetry.

### IOSFD for other types of particle radiation dosimetry

Except for photon-based RT and BT dosimetry, IOSFDs also demonstrate the potential for dosimetry in other types of therapy treatments such as PT and BNCT [73–79]. PT offers improved dose distribution compared to ionizing photon radiation. The deposited dose reaches the maximum near the end of the transporting range (the Bragg peak). However, applying SFD for PT dosimetry faces challenges whereby SFD exhibits a non-linear response to proton radiation dose at high-energy deposition of the particles within the fiber. These are due to the ionization-quenching phenomenon resulting from non-radiative de-excitation occurring under conditions of high-density energy deposition [80]; efforts have been made to find a scintillator with minimized quenching phenomenon. The IOSFDs reported in this area include a Gd_2_O_2_S:Tb-based detector and a lanthanide-doped silica scintillator-based detector [73–75]. Penner et al. [73] investigated Gd_2_O_2_S:Tb-based IOSFD for photon and proton dosimetry. For proton dose detection, the detector exhibited excellent sensitivity, signal-to-noise ratio, and reproducibility. However, further research effort is required to address the errors caused by the significant quenching.

**Fig. 10:**
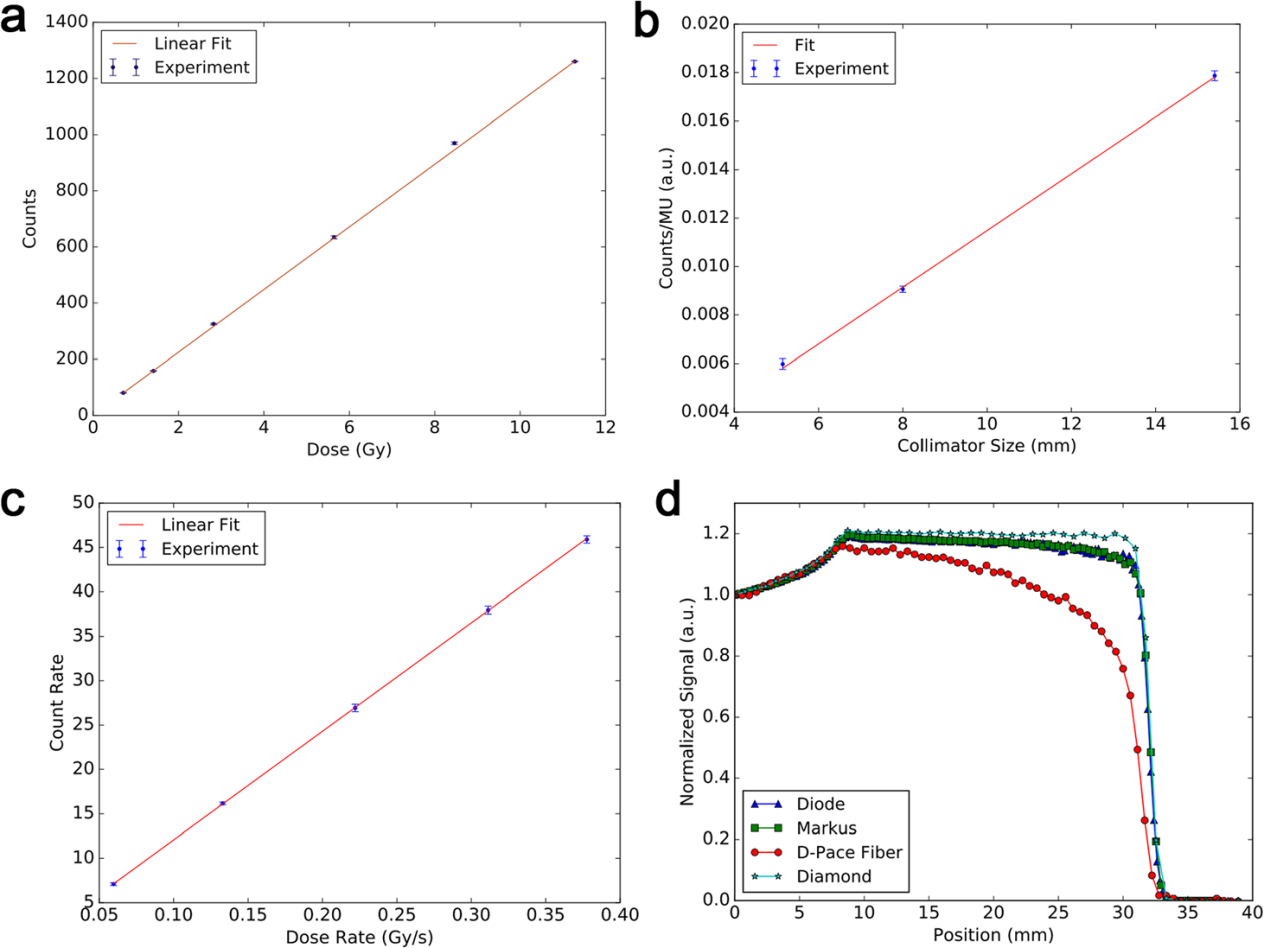
The response of a Ce-doped silica based IOSFD to a 74 MeV proton radiation source [74]. **a** Integrated counts as a function of set dose for the fiber. **b** Signal from fiber as a function of its irradiation length. **c** Count rate from the fiber as a function of dose rate. **d** Depth dose profiles for the Spread Out Bragg Peak using various dosimeters

A Ce-doped silica scintillation fiber dosimeter was tested under 74 MeV proton radiation [74]. The experiment demonstrated that the Ce-doped silica IOSFD has a good linear response to accumulated dose, irradiated dose length, and total dose rate (Fig. 10a–c). At high-density energy deposition of protons, the quenching effect is significant with a peak-to-plateau signal ratio of 2.79 for the proton raw Bragg peak profile; nevertheless, this value is still lower than most standards (≥ 3.7) indicating less severe quenching as shown in Fig. 10d. Hoehr et al. [75] reported an innovative Gd-doped amorphous silica bulk scintillator for dosimetry in PT. This Gd-doped silica IOSFD was tested with 8.2–62.9 MeV protons and 2–6 nA of extracted beam current and then compared with Ce- and Cu-doped silica IOSFDs fabricated with the same sol-gel technique. Three types of IOSFDs all exhibited strong luminescence and linear response to proton radiation. Of these, the Gd-doped silica IOSFD shows superior resolution of the Bragg peak with the lowest Birks’ constant (*k*_B_ = (0.0162 ± 0.0003) cm/MeV), which is also lower than common PSFD, indicating a significant reduced quenching. Their research demonstrated that this Gd-doped silica IOSFD could be a promising candidate for PT dosimetry, and future steps to minimize the detector size were suggested.

During the BNCT process, ^6^Li-based inorganic scintillators have been adopted to measure the neutron flux. Thermal and epi-thermal neutrons are captured by ^6^Li(n,t)α reactions after which α particles and tritons are emitted. Other scintillation components are absorbed by these secondary particles and emit light photons. Ito et al. [76] fabricated a ^6^LiF-ZnS(Ag)-based IOFD to monitor a thermal neutron flux for BCNT. Two types of probes were fabricated: one comprised a thin ZnS(Ag) film and a thin ^6^LiF film, and another comprised a wavelength-shift (WLS) fiber coated with a ZnS(Ag) film and a thin ^6^LiF film. These two probes aimed to improve gamma-neutron discrimination ability and neutron detection efficiency, respectively. Watanabe et al. [81] used a similar ^6^LiF-ZnS(Ag)-WLS combination to increase the neutron sensitivity. The effective length of the detector has a positive effect on the sensitivity. Watanabe et al. [78] reported a small Eu:LiCaAlF_6_ scintillator-based IOSFD for neutron detection in BNCT. The light yield, the light collecting efficiency, and the transparency of the scintillator were improved compared with the aforementioned ^6^LiF-ZnS(Ag)-based IOSFD. The experiment confirmed an enhancement on the gamma-neutron discrimination ability. However, the output linearity and dynamic range in real BNCT field requires further investigation.

The “Development of IOSFDs” section reviews IOSFDs with different scintillation materials and sensor configurations for dosimetry in ionizing photon RT, ^192^Ir BT, PT, and BNCT. Clinical applications still require more research to identify proper scintillation materials, scintillator fiber-coupling configurations, and correction factors as well as protocols for IOSFD for real-time in vivo dosimetry.

## Challenges of IOSFD for RTD application

Recent years have witnessed promising progress in IOSFD research, but there still remains some concern over the clinical utilization of IOSFDs for in vivo dosimetry. This section will discuss three major challenges in this research area: PDD deviation from the standard IC measurement, the angular dependence, and the Cherenkov effect.

### PDD deviation from the standard IC measurement

The PDD curve is used to measure the percentage of dose deposited in water at different depths, with respect to the point of maximum dose. The curves measured with SFDs are normally compared with standard IC. One attractive merit of PSFD is the water-equivalent property of the plastic organic scintillators and plastic optical fibers; therefore, the PSFD normally has no interference on the dose distribution. Early research on PSFD has demonstrated a good agreement with IC regarding the PDD test [15]. A maximum error of 0.3% was achieved by neglecting the Cherenkov effect when the detector was placed perpendicular to the central axis of a 10 MV X-ray radiation beam (field size 10 × 10 cm^2^). However, most of IOSFDs exhibited non-ignorable deviation of depth-dose characteristics compared to the IC measurement. To date, this remains the major impediment to the clinical application of IOSFD. For example, the Gd_2_O_2_S:Tb-based IOSFD exhibited overresponse to the X-ray (6 MV, 100 MU) when the field size is larger than 10 × 10 cm^2^ (at 1.5 cm in water equivalent material where the radiation dose reaches maximum (*D*_max_) for a 6-MV photon beam measured by the IC) [27]. The deviation increased as the field size increased. The magnitude of the deviation depends not only on the field size but also on the type of scintillator, the incident radiation beam profile, and the sub-surface depth [27, 58, 63, 82–85].

Multiple factors may give rise to the PDD deviation using IOSFD sensors compared with ICs. In the study on YVO_4_:Eu, Gd_2_O_2_S:Tb, and ZnSe(Te)-based IOSFDs, the overresponse of the IOSFD to radiation in PDD experiment was attributed to the higher *Z*_eff_ of these inorganic scintillators [59, 83, 86]. The high *Z*_eff_ results in an over-response of IOSFD with respect to water or water-equivalent material due to the secondary radiation of low energy. A recent study reported a new model investigating the overresponse phenomenon in PDD measurement with IOSFD [63]. The experiment verified that the influence of the energy effect and Cherenkov effect only contributed to a small part of PPD deviation from the IC measurement. To explain the overresponse phenomenon, the new model considers the softening of the energy spectrum of the incident X-ray/γ-ray and the secondary electrons, as well as the sensitivity of IOS to photons and secondary electrons with lower kinetic energies. As the photons penetrate into the water phantom, the mean energy of the photon beam and secondary electrons decreases due to photon interactions [87, 88]. The IOS is more sensitive to photons with lower energy in the deeper depth. The model also suggests that the scintillation efficiency of IOS corresponding to lower energetic secondary electron absorption is also higher considering the inner scintillation mechanism. This group used the shielding of the low energy particles and different beam size experiments to prove the theory. However, further Monte-Carlo simulation-based research (including a thorough consideration of the incident beam profile, secondary electrons, the radiation field size, and the probe size of the IOSFD) would be helpful to understand this theory.

This same group proposed a solution to correct the PDD curves of IOSs [85]. This method utilized the fact that different scintillators have unique PDD characteristics and near-perfect dose linearity for a fixed depth. By applying a parallel-paired fiber light guide structure where the two different scintillator materials (Gd_2_O_2_S:Tb and NaI:Tb in this case) are separately embedded in two fiber tips, the information of water depth and absorbed dose at the point of measurement can be extracted. This method provides one solution to overcome the PDD curve uncertainty and errors using IOSFD. However, the accuracy of applying this method to a clinical trial with respect to varying field sizes still needs further investigation. Overall, for the wide clinical application of IOSFD, more efforts are needed to study the cause and solution of the PDD deviation of various IOSFDs with respect to the IC measurements.

### Angular dependence

Angular dependence was also constantly mentioned in the research concerning IOSFDs [19, 59, 89, 90]. The angular dependence arises from the geometrical asymmetry of the scintillator material in the sensor tip. For better coupling with the optical fiber, the scintillation part is normally shaped in the form of cylinder geometry with a small diameter (smaller than 1 mm) and relatively long length (several millimeters; Figs. 4, 5, 6, 7, and 8). As the angle changes, photons or electrons which enter the cylindrical sensor tip from different locations and directions may have different initial energies and travel further through paths of different lengths in the scintillator material. Hence, it is reasonable to presume that the angular changes may cause a fluctuation of response to the radiation.

**Fig. 11:**
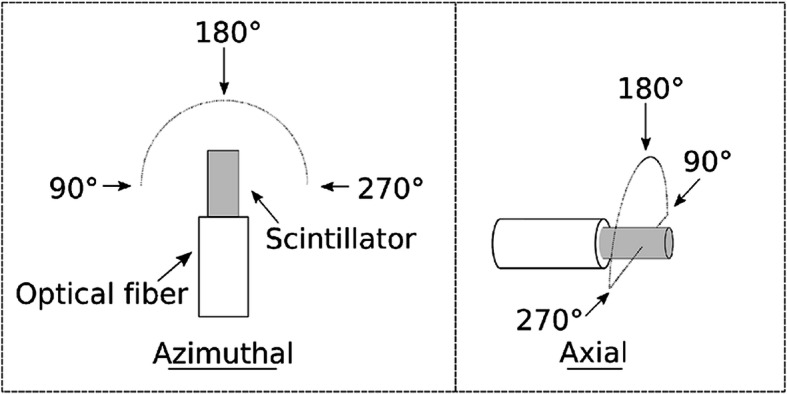
The schematic of the angler dependence measurement showing the azimuthal angular change and the axial angular change [59]. The arrows by the angle 90°, 180°, and 270° give the direction of incident radiation

Considering the geometrical symmetry, response of IOSFD to radiation with respect to angular changes along axial direction and azimuthal direction of the scintillator and optical fiber (Figs. 11) have different characteristics [59]. IOSFDs with cylindrical sensor tips normally exhibit great axial angular independence and noticeable azimuth angular dependence as shown by Qin et al. [19] and Alharbi et al. [90]. A solution to this issue was suggested from the aspect of geometry [59]. The scintillator powders were dispersed in a quasi-sphere droplet compared with a cylinder-shaped scintillator tip. The probe with this quasi-spherical geometry exhibited better azimuth angle independence with an observed variation less than 2%. Therefore, it is possible to achieve angular independence by optimizing the geometry of the scintillator material pack.

## Cherenkov effect removal techniques

The Cherenkov effect is considered as a major interference when using SFDs for dosimetry. It describes the phenomena that electromagnetic radiation is emitted when the charged particles (e.g., electrons and positrons) passes through a material with the speed exceeding the phase velocity of light in that medium. It occurs in almost every clear material (e.g., silica or plastic optical fiber) at electron energies approximately 180 keV [56]. The Cherenkov light signal covers a broadband spectrum ranging from UV to infrared wavelength. The Cherenkov radiation from the optical fiber and scintillators limits the accuracy of the dosimeter. The intensity of the Cherenkov radiation is of the same order of magnitude as the scintillation signal of the plastic scintillator, thus it must be removed if PSFD is applied [91, 92]. In IOSFD, the intensity of the scintillation signal is significantly stronger than that of the Cherenkov signal. Nevertheless, it is still necessary to remove the Cherenkov effect and only account for the scintillation signal. Here, we briefly summarize the solutions to remove the Cherenkov effect including examples of both inorganic and organic scintillator-based SFDs.

Studies on the generation of Cherenkov radiation have been carried out for both silica and plastic optical fiber [62, 93–95]. At least four different methods have been devised to eliminate the Cherenkov effect. These methods either remove Cherenkov physically or take advantage of the characteristic differences of the scintillation and Cherenkov radiation (such as the spectrum and the rise-decay time).

The first method, presented by Beddar et al. [14], removed the Cherenkov background signal by employing a parallel optical fiber located close to the signal fiber that coupled with a piece of scintillator. The light signal detected in the second fiber is an approximation (or a reference) of the Cherenkov background in the signal fiber. The scintillation signal was corrected by subtracting this background signal detected by the reference fiber. The limitation of this method is that any positional differences between the two fibers will introduce errors [95], and it increases the size of the dosimeter.

The second method exploits the spectral difference between the scintillation light and the Cherenkov radiation [61]. Importantly, the intensity of the Cherenkov spectrum is proportional to λ^−3^ (where λ is the wavelength of the Cherenkov light), and the emission peak is in the blue/green light region. By using scintillation coupled with a longer wavelength emission (e.g., red emission peak) and filtering out the light with a shorter wavelength, the Cherenkov emission was decreased from 6.5 to 2.8% of the scintillator’s signal. Still, the Cherenkov signal cannot be totally eliminated using this method, as Cherenkov light spectrum is continuous and comprises photons with long wavelength. Later, Fontbonne et al. [96] demonstrated that by measuring the absolute dose and the intensities of green and blue emission at two different locations, the Cherenkov background signal can be, in principle, eliminated using linear equations to calculate the contribution of the Cherenkov signal and the scintillation signal. Further research regarding the correction of the Cherenkov effect based on this spectral discrimination method was presented by Frelin et al. [97], Guillot et al. [21], and Ishikawa et al. [98].

The third method, which has been mentioned in the “Basic physical properties of scintillators” section, was presented by Clift et al. [20]. It is a temporal method, and it relies on the fact that the Cherenkov emission is a prompt process, whereas scintillation is a delayed process. The Cherenkov signal could be reduced by reading the signal from the dosimeter between the linac pulses when the Cherenkov radiation was decayed to almost zero. An organic scintillator BC-444G with a long decay constant (264 ns) was used to maximize the amount of scintillation light emitted in between the linac pulses, and a sampling time of 700–705 ns was adopted. This setup successfully eliminated most of the Cherenkov and fluorescent radiation.

The last method, proposed by Lambert et al. [91], corrected the Cherenkov signal “physically” by removing the media where the Cherenkov signal is produced. In this case, the fiber core exposed in radiation was removed. The experiment used an air core light guide to transport the light from the scintillator to the light detector. This theoretically eliminates the generation of Cherenkov light at its source and thus provides a novel solution to the Cherenkov effect.

## Conclusion

This work reviewed the work on IOSFDs for medical radiation dosimetry. First, some basic knowledge regarding scintillator material was introduced. The fundamentals of IOSs were reviewed in the “Fundamentals” section in terms of overall scintillation efficiency, decay time, mass attenuation coefficients, and energy dependence. Dense IOSs with high photon attenuation coefficient and scintillation efficiency are favorable for the application of IOSFD for low-dose rate in vivo dosimetry. The “Development of IOSFDs” section detailed the development of IOSFDs by applying different scintillator materials and configurations. Research concerning embedded-structure IOSFDs and sensor tip modification certifies that by optimizing the scintillator coupling method, it is possible to improve the coupling efficiency between the scintillator and the optical fiber. Furthermore, various IOSs with the bright red emission maximum provide convenience to suppress the Cherenkov effect and stem effect in the optical fiber using a spectrum filtering method. IOSFD also offers promising potential for dosimetry in BT, PT, and BNCT, but problems like the quenching phenomenon must be addressed. The response characteristics of different IOSs to radiation dose from various charged particle sources should be investigated in the future. In the “Challenges of IOSFD for RTD application” section, challenges for IOSFD development including PDD deviation from standard IC measurement and angular dependence were discussed. In the “Cherenkov effect removal techniques” section, typical Cherenkov removal techniques were summarized. Clinical applications still require more research effort to identify a proper scintillator material, scintillator-fiber coupling configuration, and correction factor; protocols using IOSFD for in vivo and real-time dosimetry are also still needed. Nevertheless, it is reasonable to expect more use of IOSFDs for in vivo real-time RTD considering their good linearity and repeatability, high sensitivity, and convenience for stem effect removals.

## Data Availability

The datasets generated during the photon attenuation coefficient analyses as shown Fig. 2 are available from XCOM: Photon Cross Sections Database on the website https://www.nist.gov/pml/xcom-photon-cross-sections-database

## Abbreviations

BT: Brachytherapy
BNCT: Boron neutron capture therapy
Z_eff: Effective atomic number
IOSFD: Inorganic scintillator-based optical fiber dosimeter
IOS: Inorganic scintillators
IMRT: Intensity-modulated radiotherapy
IC: Ion chamber
linac: Linear accelerator
max: Maximum
OS: Organic scintillator
PDD: Percent depth dose
PSFD: Plastic scintillator-based optical fiber dosimeter
PMMA: Polymethyl methacrylate
PT: Proton therapy
QA: Quality assurance
RT: Radiotherapy
RTD: Radiotherapy dosimetry
SFD: Scintillator-based optical fiber dosimeter
SABR: Stereotactic ablative radiotherapy
UV/vis: Ultraviolet/visible
WLS: Wavelength-shift
